# Determination of Tenacissoside G, Tenacissoside H, and Tenacissoside I in Rat Plasma by UPLC-MS/MS and Their Pharmacokinetics

**DOI:** 10.1155/2023/4747771

**Published:** 2023-09-28

**Authors:** Fan Chen, Yizhe Ma, Ying Cui, Wanhang Wang, Chenchen Mei, Jingjing Nie, Congcong Wen, Xiuwei Shen, Xuzhao Zhou

**Affiliations:** ^1^Ruian People's Hospital, The Third Affiliated Hospital of Wenzhou Medical University, Wenzhou, China; ^2^Laboratory Animal Centre, Wenzhou Medical University, Wenzhou, China; ^3^The Molecular Neuropharmacology Laboratory and the Eye-Brain Research Center, The State Key Laboratory of Ophthalmology, Optometry and Vision Science, School of Ophthalmology & Optometry and Eye Hospital, Wenzhou Medical University, Wenzhou, China

## Abstract

An ultra-performance liquid chromatography/tandem mass spectrometry (UPLC-MS/MS) method was developed for the determination of tenacissoside G, tenacissoside H, and tenacissoside I in rat plasma. The rat plasma was treated with liquid-liquid extraction using ethyl acetate. The determination was performed on the UPLC HSS T3 column (50 mm × 2.1 mm, 1.8 *μ*m) with a mobile phase consisting of acetonitrile-water (containing 0.1% formic acid) and gradient elution at a flow rate of 0.4 mL/min. Electrospray (ESI) positive ion mode detection and multireaction monitoring (MRM) quantitative analysis were performed. A total of 36 rats were given tenacissoside G, tenacissoside H, and tenacissoside I, respectively, orally (5 mg/kg) and intravenously (1 mg/kg), with 6 rats in each group, to evaluate the pharmacokinetic difference of tenacissoside G, tenacissoside H, and tenacissoside I in rats. The calibration curves showed good linearity in the range of 5–2000 ng/mL, where *r* was greater than 0.99. The results of precision, accuracy, recovery, matrix effect, and stability met the requirements of biological sample detection methods. The established UPLC-MS/MS method was successfully applied to pharmacokinetic studies of tenacissoside G, tenacissoside H, and tenacissoside I, and the bioavailability was 22.9%, 89.8%, and 9.4%, respectively.

## 1. Introduction

*Marsdenia tenacissima*, also known as *glaucescent fissistigma* root, *Rosa banksiae* f. *lutea* (Lindl.) Rehd, etc., is the dry stem and rattan of *Marsdenia tenacissima* (Roxb) Wight et Am., a plant belonging to the family Asclepiadaceae [1–3], which was originally published in the Herbal Medicines of Southern Yunnan and is now published in the Chinese Pharmacopoeia 2010 edition [4, 5]. It is mainly distributed in Guizhou, Yunnan, Sichuan, Guangxi, and other places. *Marsdenia tenacissima* tastes bitter and slightly cold. It has the effects of clearing heat and detoxifying, relieving cough, and asthma, dispersing knots and relieving pain, and fighting cancer. The series of preparations made from its single medicinal material are widely used in clinics [6–8].

The roots, stems, and leaves of *Marsdenia tenacissima* can be used as medicine. Its effective components are steroidal glycosides, alkaloids, and polysaccharides [9–11]. Its active antitumor components are mainly steroidal components. It is reported that *Marsdenia tenacissima* polysaccharides and some fat-soluble components also have antitumor effects [12]. At present, dozens of steroids have been isolated from this plant. The C21 steroidal glycosides are mostly white crystals or powder, which mainly exist in the plants of Asclepiadaceae. The main antitumor active component of *Marsdenia tenacissima* is C21 steroidal glycosides [13]. Polysaccharide and some fat-soluble components also have antitumor effects. At present, more than 40 kinds of C21 steroidal glycosides have been isolated from the plant, which are a class of compounds formed by a class of steroidal derivatives of glycosides and 2-deoxysugars, and the sugar chain contains up to 6 sugars. These compounds contain a variety of aglycones with different structures [8, 9], of which there are 6 main configuration aglycones. In *vivo* pharmacokinetics of tenacissoside H and tenacissoside I have been reported in literature [14–16], but bioavailability has not been reported.

High-performance liquid chromatography tandem mass spectrometry (LC-MS/MS) technology has the advantages of high sensitivity, low detection limit, and small sample consumption and is widely used in drug analysis of chemical composition, drug metabolism, and impurity identification [17, 18]. UPLC, which columns with small particle sizes and under ultra-high pressure, maintains the basic principles of a traditional HPLC system but demonstrates improved separation efficiency and speed [19, 20].

Therefore, this study was to establish an UPLC-MS/MS for the determination of tenacissoside G, tenacissoside H, and tenacissoside I in rat plasma and study the pharmacokinetics and bioavailability to provide a scientific experimental basis for the basic research of clinical pharmacy.

## 2. Experimental

### 2.1. Reagents and Animals

**Figure 1:**
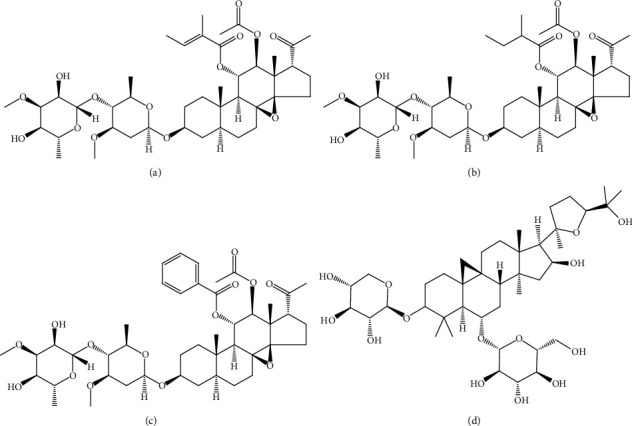
Chemical structures of tenacissoside G (a), tenacissoside H (b), tenacissoside I (c), and Astragaloside IV (d).

Tenacissoside G, tenacissoside H, tenacissoside I, and astragaloside IV (internal standard) (purity ≥98%, Figure 1) were all purchased from Chengdu Master Pharmaceutical Co., Ltd. Acetonitrile and methanol in chromatographic purity were purchased from Merck. Ultra-pure water (resistance >18 mΩ) was prepared by the Milli-Q purification system in the United States. Sprague–Dawley (SD) rats (220–250 g) were from the Animal Experimental Center of Wenzhou Medical University.

### 2.2. Instrument Condition

A Waters XEVO TQ-S microtriple quadrupole series mass spectrometer was used for the detection of tenacissoside G, tenacissoside H, and tenacissoside I.

Chromatographic conditions were as follows: UPLC HSS T3 column (50 mm × 2.1 mm, 1.8 *μ*m) and column temperature set at 40°C. The mobile phase was acetonitrile-water (containing 0.1% formic acid) with gradient elution at a flow rate of 0.4 mL/min and elution time of 6 min. 0–0.2 min, acetonitrile 10%; 0.2–2.4 min, acetonitrile 10%–75%; 2.4–5.0 min, acetonitrile 75%–90%; 5.0–5.1 min, acetonitrile 90%–10%; and 5.1–6.0 min, acetonitrile 10%.

Mass spectrometry conditions: nitrogen as conical gas (50 L/h) and desolvated gas (900 L/h); capillary voltage set at 2.5 kV; ion source temperature at 150°C; and desolvent temperature at 450°C. ESI positive ion mode detection and MRM were used for quantitative analysis: *m*/*z* 815.5 ⟶ 755.5 for tenacissoside G (cone voltage 96 v, collision voltage 26 v), *m*/*z* 817.4 ⟶ 757.5 for tenacissoside H (cone voltage 96 v, collision voltage 40 v), *m*/*z* 837.4 ⟶ 777.5 for tenacissoside I (cone voltage 86 v, collision voltage 30 v), and *m*/*z* 785.4 ⟶ 143.0 for astragaloside IV (cone voltage 6 v, collision voltage 46 v).

### 2.3. Standard Curve

Tenacissoside G, tenacissoside H, tenacissoside I, and astragaloside IV reserve solution (500 *μ*g/mL) were prepared with methanol, respectively. Tenacissoside G, tenacissoside H, and tenacissoside I working solution was obtained by diluting the reserve solution with methanol. Both the reserve solution and working solution were stored at 4°C. Appropriate amount of tenacissoside G, tenacissoside H, and tenacissoside I working solution was added to the blank rat plasma, and then the tenacissoside G, tenacissoside H, and tenacissoside I in rat plasma were 5, 10, 20, 50, 100, 200, 500, 1000, and 2000 ng/mL. Three quality control (QC) samples with plasma concentrations (8, 180, and 1800 ng/mL) were prepared by the same method.

### 2.4. Sample Handling

A plasma sample of 100 *μ*L was added to a 1.5 mL microcentrifuge tube, then 10 *μ*L of astragaloside IV (1.0 *μ*g/mL) and 1.0 mL ethyl acetate were added, vortex was mixed for 1.0 min, and it was centrifuged (13,000 rpm, 4°C, 5 min). The organic phase was transferred into another tube and evaporated to dryness at 40°C under a gentle stream of nitrogen. The residue was reconstituted in 100 *μ*L methanol and centrifuged at 13,000 rpm for 5 min. The supernatant was pipetted to an auto sampler vial, and 2 *μ*L was injected into the UPLC-MS/MS for analysis.

### 2.5. Pharmacokinetic Study

Tenacissoside G, tenacissoside H, and tenacissoside I were given sublingual intravenous administration (iv) of 1 mg/kg and oral administration (po) 5 mg/kg, respectively, with 6 rats in each group, for a total of 36 rats. All experimental procedures and protocols were approved by the Animal Care Committee of Wenzhou Medical University (xmsq 2023-0689). The 0.4 mL blood was collected from the caudal vein at 0.083 3, 0.5, 1, 2, 3, 4, 6, and 8 h for tenacissoside G; 0.083 3, 0.5, 1, 2, 3, 4, 6, 8 and 12 h for tenacissoside H; and 0.083 3, 0.5, 1, 2, 3, 4, and 6 h for tenacissoside I, collected in heparinized test tubes and centrifuged at 13000 r/min for 10 min. 100 *μ*L of the plasma was then transferred to a new 1.5 mL microcentrifuge tube and held at −80°C prior to analysis. Pharmacokinetic parameters were statistically calculated using the pharmacokinetic software (DAS 2.0 version).

## 3. Result

### 3.1. Selectivity

**Figure 2:**
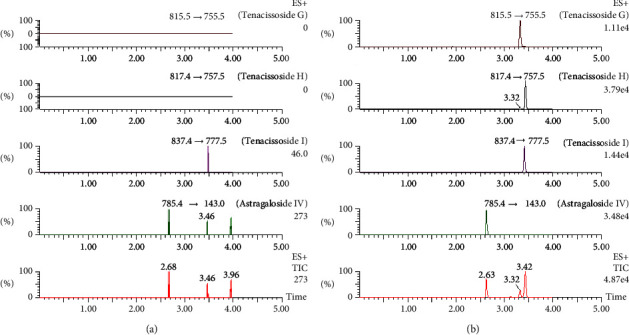
UPLC-MS/MS of tenacissoside G, tenacissoside H, tenacissoside I, and astragaloside IV in rat plasma: (a) blank plasma and (b) blank plasma spiked into tenacissoside G, tenacissoside H, tenacissoside I, and astragaloside IV.

The retention times of tenacissoside G, tenacissoside H, tenacissoside I, and astragaloside IV were 3.32, 3.42, 3.41, and 2.63 min, as shown in Figure 2, respectively. The optimized gradient elution procedure was used to isolate tenacissoside G, tenacissoside H, and tenacissoside I, effectively, and no interference of endogenous components was observed in the retention time of tenacissoside G, tenacissoside H, and tenacissoside I. This method has good selectivity.

### 3.2. Standard Curve

The calibration curves of tenacissoside G, tenacissoside H, and tenacissoside I in rat plasma showed good linearity in the range of 5–2000 ng/mL, with *r* greater than 0.99. The typical regression equation of the tenacissoside G in rat plasma was as follows: *y*1 = 0.0045*x*1 + 0.0102 (*r* = 0.9976), *x*1 was the concentration of tenacissoside G in plasma, and *y*1 was the ratio of tenacissoside G peak area to the internal standard. The typical regression equation for tenacissoside H in rat plasma was *y*2 = 0.0046*x*2 + 0.0022 (*r* = 0.9986), where *x*2 was the concentration of tenacissoside H in plasma, and *y*2 was the ratio of the tenacissoside H peak area to the internal standard. The typical regression equation for tenacissoside I in rat plasma was *y*3 = 0.0020*x*3 + 0.0097 (*r* = 0.9977), *x*3 was the concentration of tenacissoside I in plasma, and *y*3 was the ratio of tenacissoside I peak area to internal standard. The lower limit of quantitation of tenacissoside G, tenacissoside H, and tenacissoside I in rat plasma was 5 ng/mL, and the detection limit was 1.5 ng/mL.

### 3.3. Precision, Accuracy, Recovery, and Matrix Effect

The intraday and interday precision of tenacissoside G was within 10%, the accuracy was 90% to 111%, the recovery was over 92%, and the matrix effect was in the range of 94% to 109% (Table 1).

The intraday and interday precision of te nacissoside H was within 13%, the accuracy was 88% to 115%, the recovery was above 88%, and the matrix effect was in the range of 101% to 108% (Table 1).

The intraday and interday precision of tenacissoside I was within 15%, the accuracy was 88% to 110%, the recovery was above 80%, and the matrix effect range was 91% to 99% (Table 1).

### 3.4. Stability

The plasma samples of rats were stored in an automatic injector for 2 h, pretreated, and placed at room temperature for 24 h. The plasma samples underwent three freeze-thawing cycles, and the stability test was conducted at −20°C for 30 days. The accuracy of tenacissoside G was 88%–112%; RSD was within 13%. The accuracy of tenacissoside H was 88%–111%; RSD was within 15%. The accuracy of tenacissoside I was 88%–111%; RSD was within 15% (Table 2). The results indicated that tenacissoside G, tenacissoside H, and tenacissoside I were stable.

### 3.5. Pharmacokinetic Study

**Figure 3:**
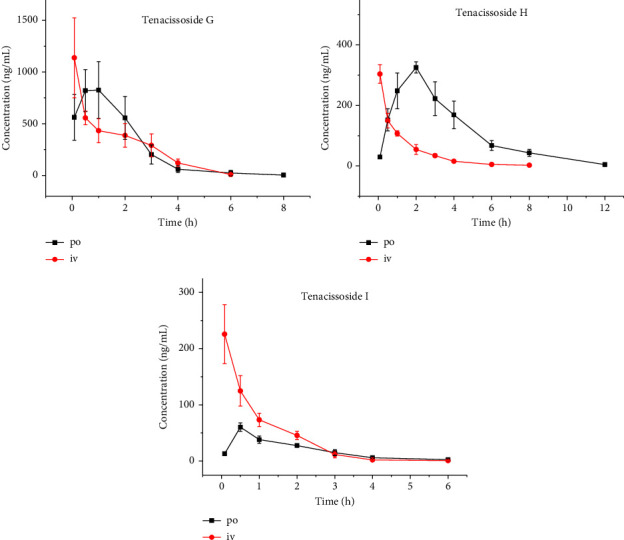
The concentration-time curve of rats after intravenous (iv, 1 mg/kg) and oral (po, 5 mg/kg) administration of tenacissoside G, tenacissoside H, and tenacissoside I (*n* = 6).

The concentration-time curves of tenacissoside G, tenacissoside H, and tenacissoside I in rat plasma are shown in Figure 3. The main pharmacokinetic parameters are listed in Table 3, and oral bioavailability was 22.9%, 89.8%, and 9.4%, respectively.

## 4. Discussion

In order to obtain the best mass spectrum conditions, the positive and negative ion modes were used for monitoring. The responses of tenacissoside G, tenacissoside H, and tenacissoside I were higher in the positive ion mode than the negative ion modes. After optimizing various parameters, the mass spectrum parameters can meet the requirements of accurate quantification of effective substances in biological samples. Through the standard sample, the capillary voltage and the collision energy were optimized.

The commonly used biological sample pretreatment methods were liquid-liquid extraction (LLE) [21–24], solid-phase extraction (SPE) [25, 26], and protein precipitation method (PPT). The SPE method has complicated operation steps and a high extraction column price. LLE could efficiently extract target substances, especially for low-concentration samples, with higher extraction efficiency than PPT. LLE and PPT methods were tried in this work, and LLE with ethyl acetate was found to be a better extraction efficiency (around 90%) than PPT with acetonitrile (around 60%).

During quantitative analysis, it is necessary to add an internal standard substance of known concentration as a quantitative reference for the compounds to be measured in the sample. The internal standard substance should have similar physical and chemical properties to the compound to be tested, and be stable in the sample, and be easy to detect and quantify. Commonly used internal standard substances include isotope-labeled compounds and structural analogues. Astragaloside IV has similar physical and chemical properties to tenacissoside, and it was selected as the internal standard.

Zhao et al. studied the plasma concentration and pharmacokinetic process of tenacissoside A in rats by the LC-MS/MS method [27]. Medroxyprogesterone acetate was used as the internal standard. The oral bioavailability of the drug was low (2.6%), the elimination was faster, and the first-pass effect was obvious. Li established a LC-MS/MS method for simultaneous determination of garcinia extract in rat plasma samples of tenacissoside B, tenacissoside H, tenacissoside I, caffeic acid, cryptochlorogenic acid, chlorogenic acid, and neochlorogenic acid [16]. Digoxin was used as an internal standard reference. Zeng et al. have developed a LC-MS/MS method for simultaneous determination of three isomerized gestrins (17*β*-tenacigenin B, tenacigenine B, and tenacigenine A) and their corresponding glycosides (tenacissoside A and tenacissoside B) in rat plasma [14]. After dexamethasone acetate was added as an internal standard, a simple liquid-liquid extraction technique was used. This method was successfully applied to the pharmacokinetic study after intravenous injection of Xiao-Ai-Ping in rats. However, these methods did not study the bioavailability of tenacissoside B, tenacissoside H, and tenacissoside I.

## 5. Conclusion

In this study, the UPLC-MS/MS technique was established for the determination of tenacissoside G, tenacissoside H, and tenacissoside I in rat plasma in the range of 5–2000 ng/mL. The rat plasma was treated with liquid-liquid extraction using ethyl acetate, and astragaloside IV was used as internal standard. The selectivity, linearity, precision, accuracy, recovery, and stability of this method have been verified, and it has been applied to the pharmacokinetic study of tenacissoside G, tenacissoside H, and tenacissoside I in rats, and the bioavailability was calculated to be 22.9%, 89.8%, and 9.4%, respectively.

## Figures and Tables

**Table 1:** Accuracy, precision, matrix effect, and recovery of tenacissoside G, tenacissoside H, and tenacissoside I in rat plasma.

Compound	Concentration (ng/mL)	Accuracy (%)		Precision (RSD%)		Matrix effect (%)	Recovery (%)
		Intraday	Interday	Intraday	Interday		
Tenacissoside G	5	90.6	110.5	10.0	10.0	108.5	92.9
	8	108.8	99.0	8.4	4.8	94.5	92.2
	180	99.4	105.5	5.3	9.3	102.3	97.3
	1800	104.4	104.9	8.1	4.8	99.8	94.4

Tenacissoside H	5	91.1	97.9	13.0	11.8	106.9	97.2
	8	88.2	109.8	10.2	9.1	101.8	96.9
	180	100.9	92.8	11.0	8.3	102.1	90.6
	1800	96.2	114.5	6.8	2.0	107.8	88.6

Tenacissoside I	5	95.3	88.8	8.8	14.1	92.8	83.5
	8	93.2	105.8	7.2	6.4	91.3	83.0
	180	109.8	101.6	10.0	10.7	94.1	80.6
	1800	101.3	92.9	9.0	7.8	98.5	85.8

**Table 2:** Stability of tenacissoside G, tenacissoside H, and tenacissoside I in rat plasma.

Compound	Concentration (ng/mL)	Autosampler (4°C, 12 h)		Ambient (2 h)		−20°C (30 d)		Freeze-thaw	
		Accuracy	RSD	Accuracy	RSD	Accuracy	RSD	Accuracy	RSD
Tenacissoside G	8	102.1	11.3	104.9	12.9	106.8	12.9	100.0	9.1
	180	97.8	6.7	95.0	4.9	99.9	1.4	111.3	11.5
	1800	100.2	2.2	100.1	8.9	93.4	5.7	88.7	12.3

Tenacissoside H	8	102.6	9.5	102.3	7.8	102.2	12.3	94.8	10.9
	180	101.5	4.7	95.5	11.8	88.2	3.6	110.9	14.5
	1800	95.9	7.5	102.2	7.9	109.6	3.9	94.2	7.1

Tenacissoside I	8	94.8	5.1	105.5	10.9	90.4	14.3	88.0	8.9
	180	103.0	4.5	95.3	5.0	103.1	11.2	109.2	13.0
	1800	102.2	8.5	99.2	8.9	106.6	2.8	102.8	13.4

**Table 3:** Main pharmacokinetic parameters after intravenous (IV, 1 mg/kg) and oral (PO, 5 mg/kg) administration of tenacissoside G, tenacissoside H, and tenacissoside I in rats.

Compound	Group	AUC_(0-t)_ (ng/mL·h)	AUC_(0-∞)_ (ng/mL·h)	*t*_1/2z_ (h)	CL_z/F_ (L/h/kg)	*V*_z/F_ (L/kg)	*C*_max_ (ng/mL)
Tenacissoside G	po	2037.0 ± 630.4	2046.0 ± 639.2	0.9 ± 0.3	2.7 ± 0.9	3.6 ± 1.6	900.2 ± 246.3
	iv	1778.5 ± 419.6	1801.4 ± 418.2	0.8 ± 0.3	0.6 ± 0.2	0.7 ± 0.3	1137.1 ± 386.1

Tenacissoside H	po	1336.5 ± 146.1	1359.8 ± 127.7	1.8 ± 0.6	10.1 ± 4.0	325.5 ± 18.2	1336.5 ± 146.1
	iv	361.2 ± 35.7	364.6 ± 36.5	1.3 ± 0.1	5.0 ± 0.5	303.7 ± 30.9	361.2 ± 35.7

Tenacissoside I	po	113.0 ± 8.5	116.9 ± 9.9	1.1 ± 0.3	43.0 ± 3.8	69.6 ± 16.0	60.3 ± 7.7
	iv	239.5 ± 28.9	240.3 ± 28.8	0.7 ± 0.1	4.2 ± 0.6	4.3 ± 0.8	225.8 ± 52.4

## Data Availability

The data used to support the findings of this study are included within the article.
